# Frequency-domain vs continuous-wave near-infrared spectroscopy devices: a comparison of clinically viable monitors in controlled hypoxia

**DOI:** 10.1007/s10877-016-9942-5

**Published:** 2016-10-24

**Authors:** David James Davies, Michael Clancy, Daniel Lighter, George M. Balanos, Samuel John Edwin Lucas, Hamid Dehghani, Zhangjie Su, Mario Forcione, Antonio Belli

**Affiliations:** 1 National Institute for Health Research Surgical Reconstruction and Microbiology Research Centre (NIHR SRMRC), University Hospitals Birmingham NHS Foundation Trust, Heritage Building (Old Queen Elizabeth Hospital), Edgbaston, Birmingham, B15 2TH UK; 2 Department of Neurosurgery, University Hospitals Birmingham NHS Foundation Trust, Birmingham, UK; 3 PSIBS Doctoral Training Centre, University of Birmingham, Birmingham, UK; 4 School of Chemistry, University of Birmingham, Birmingham, UK; 5 School of Sport, Exercise and Rehabilitation Sciences, University of Birmingham, Birmingham, UK; 6 School of Clinical and Experimental Medicine, University of Birmingham, Birmingham, UK

**Keywords:** Head injury, Cerebral blood flow, Frequency-domain near-infrared spectroscopy, Continuous-wave near-infrared spectroscopy

## Abstract

The Near-infrared spectroscopy (NIRS) has not been adopted as a mainstream monitoring modality in acute neurosurgical care due to concerns about its reliability and consistency. However, improvements in NIRS parameter recovery techniques are now available that may improve the quantitative accuracy of NIRS for this clinical context. Therefore, the aim of this study was to compare the abilities of a continuous-wave (CW) NIRS device with a similarly clinically viable NIRS device utilising a frequency-domain (FD) parameter recovery technique in detecting changes in cerebral tissue saturation during stepwise increases of experimentally induced hypoxia. Nine healthy individuals (6M/3F) underwent a dynamic end-tidal forced manipulation of their expiratory gases to induce a stepwise induced hypoxia. The minimum end-tidal oxygen partial pressure (EtO_2_) achieved was 40 mm Hg. Simultaneous neurological and extra-cranial tissue NIRS reading were obtained during this protocol by both tested devices. Both devices detected significant changes in cerebral tissue saturation during the induction of hypoxia (CW 9.8 ± 2.3 %; FD 7.0 ± 3.4 %; Wilcoxon signed rank test *P* < 0.01 for both devices). No significant difference was observed between the saturation changes observed by either device (*P* = 0.625). An observably greater degree of noise was noticed in parameters recovered by the FD device, and both demonstrated equally variable baseline readings (Coefficient of variance 8.4 and 9.7 % for the CW and FD devices, respectively) between individuals tested. No advantageous difference was observed in parameters recovered from the FD device compared with those detected by CW.

## Background

Near-infrared spectroscopy (NIRS) represents a brain monitoring modality with many inherent advantages over available alternatives, not least its non-invasive nature, ease of application and minimal inter-observer/operator variability. Although not yet considered sufficiently accurate or consistent in its observations to be utilised within the context of brain injury or acute neurosurgical care [1, 2], the technology is commonly utilised in other clinical contexts that have potential implications on brain perfusion (e.g., cardiac surgery).

**Fig. 1:**
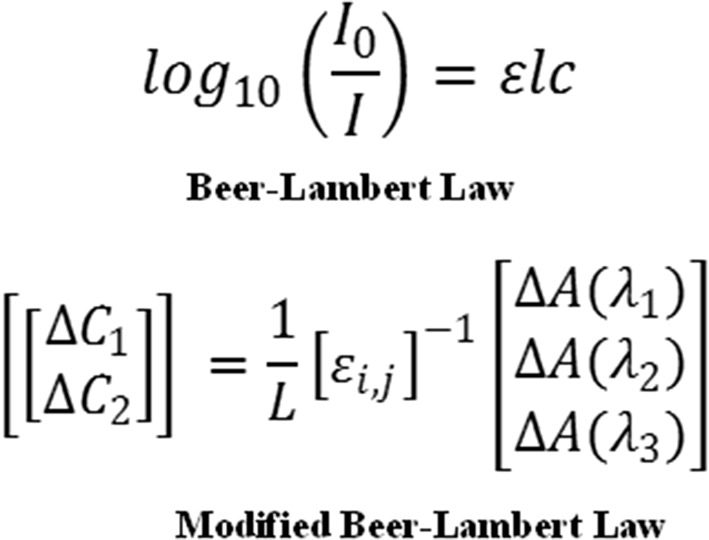
Beer–Lambert concept

Since its introduction as a clinically available monitoring modality, considerable progress has been made in technology by which the NIRS parameters are recovered [3, 4], with parameter recovery techniques evolving from those dependant solely on continuously emitted light (continuous-wave) to those incorporating frequency modulation (frequency-domain) and time of flight data (time-domain). Continuous-wave (CW) devices rely on specific assumptions within an algorithmic reconstruction, a modified Beer–Lambert principle (Fig. 1), based on the function of wavelength only regarding light scattering through the tissue medium containing chromophores (light absorbing substances). These basic principles critically assume that the degree of scatter within all areas of the field of NIRS acquisition is not only homogenous but also fixed. These assumptions are critical in the calculation of the recovered NIRS parameters, as it has been clearly demonstrated that modification of the reconstruction algorithm can produce very different results from identical raw NIRS data [5]. In addition to the reliance on assumptions made on scatter, another critical shortcoming of the CW is the assumed differential path length (L), which represents the estimated distance travelled by the light. This estimate fundamentally limits the accuracy of any interpretation of the output parameters.

One reason for the vast majority of clinical investigations employing CW-NIRS at the point of patient care is the significant cost benefit over more advanced commercially available methods of NIRS parameter recovery [6]. In order to improve the validity of CW-NIRS, multiple algorithmic manipulations have been attempted to accurately resolve NIRS parameters from a specific depth and therefore isolate light that has followed a specific path through the biological tissue. These resolution techniques or spatially resolved spectroscopy (SRS) have been adopted widely to enhance the output parameters of multiple CW-NIRS devices, most notably the Suzuki SRS concept [7] as implemented by Hamamatsu in their NIRO device. Although by no means the only SRS methodology, it serves as an excellent illustration of how parameters can be manipulated to improve the depth resolution of raw data obtained from CW devices.

**Fig. 2:**
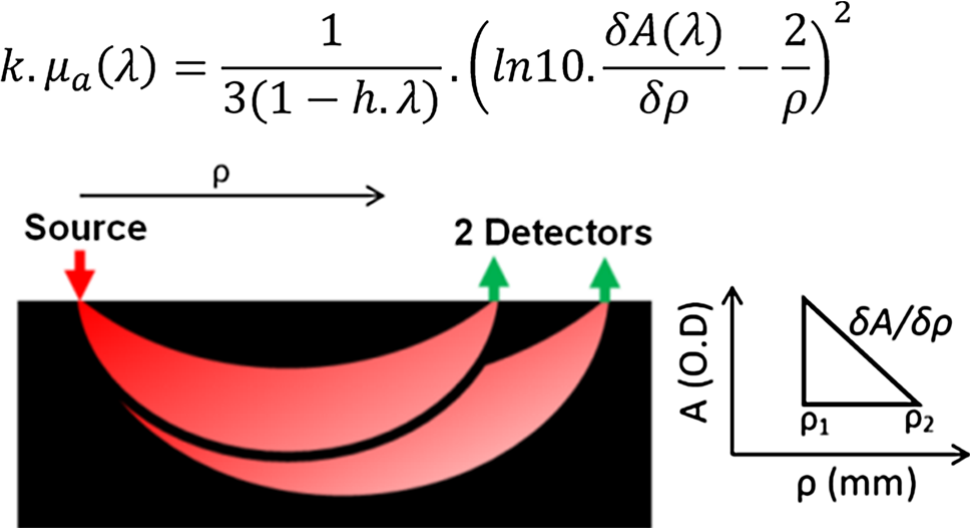
Suzuki SRS concept illustrated

Briefly, this SRS analytical solution assumes that the plate of tissue is a semi-infinite space in either direction and incorporates the horizontal distance between the light source, the light detector (*ρ*) and the attenuation of the light received at the detector (A). In a NIRS array where a single source of light arrives at 2 separate detectors (each a specific distance from the source; e.g., 38 and 42 mm) the gradient derived from the difference in attenuation arriving at each detector and the distance between each detector (δA/δρ) is utilised in order to isolate the specific portion of the light detected that has passed through tissue of the specified depth (dependant on the source detector separation (Fig. 2).

**Fig. 3:**
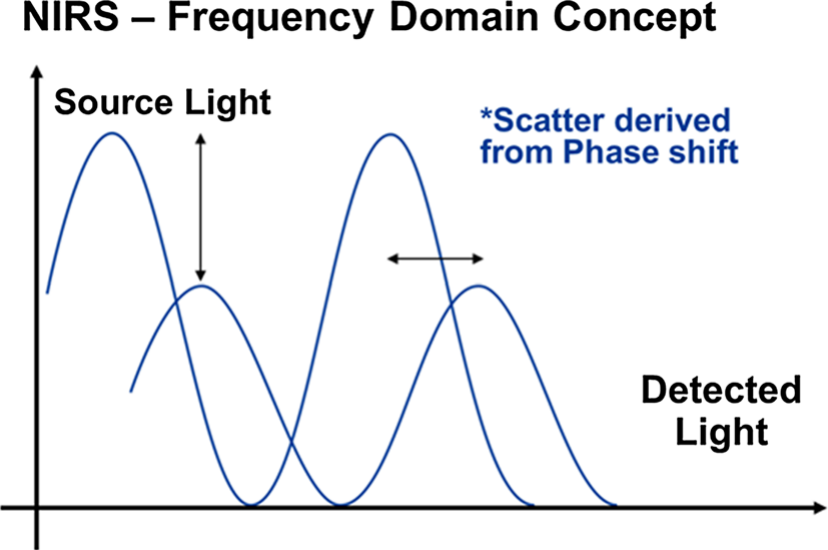
Frequency-domain concept

Frequency-domain (FD) NIRS is a progression of the CW concept that measures both light intensity attenuation and phase shift, since the intensity of the light emitted from these devices is modulated at a particular frequency. Specifically, observations regarding the shift in phase allow derivation of a more tissue specific value (quantification) of the degree of light scatter; hence the magnitude of this phenomenon is no longer assumed [8]. Theoretically, this enhanced technique allows more consistent quantitative measurements to be derived from biological tissue, particularly in the case of cerebral NIRS where a critical resolution of output parameters from a specific depth is required (Fig. 3).

Both of these techniques rely on a number of assumptions, and particularly in the case of CW devices (including those incorporating SRS parameter processing) this may contribute to superficial tissue perfusion adversely affecting the accuracy of these devices within the clinical arena [9]. Currently no clinical investigations exist to our knowledge quantifying the effect of superficial tissue blood flow on an FD NIRS device, although studies in healthy individuals have demonstrated this and made suggestions as to how to minimise its impact [10]. However even with its potential quantification of scatter within tissues this measurement is still ‘en-block’ (not depth specific) and will therefore be theoretically influenced by superficial tissue activity.

The focus of this investigation is aimed at clinically viable devices, i.e., devices that are readily available, cost effective, usable by non-specialist staff, and have appropriate approval for use by the relevant regulatory bodies. Within the context of acute clinical care (particularly traumatic brain injury), any such monitoring tool should be easily applied and removed with minimal preparation. Focusing our investigation on devices which meet these criteria maximises the translatability of any findings.

Absolute changes in arterial partial pressure of oxygen (PaO_2_) and haemoglobin (Hb) saturation (SaO_2_) vary between individuals due to a variety of factors (e.g. variable compensatory reserves and baseline). Healthy individuals undergoing induced hypoxia (e.g. at high altitude or simulated high altitude) have been shown to vary in terms of their saturation and blood gas composition. Lucas et al. [11] demonstrated a PaO_2_ standard deviation of between 11 mmHg (9.5 % of total) at sea level and 3 mmHg (7 % of total) at high altitude (5050 m) with SaO_2_ demonstrating a standard deviation of 0.5 % at sea level and approximately 3 % at altitude. A similar distribution of variation was demonstrated in arterial partial pressure of carbon dioxide (PaCO_2_). We can infer from this that baseline variability (and standard deviation) in cerebral saturation at sea level and simulated altitude as observed by a NIRS device of sufficient quantitative accuracy to be utilised clinically should be within a similar order.

## Aims and study design

In this study we aimed to simulate cerebral tissue hypoxia (a physiological change pertinent to Traumatic Brain Injury (TBI) pathology) in order to compare output parameters recorded simultaneously from clinically viable CW and FD cerebral NIRS devices. Our primary hypothesis being that the inherent advantages of a system that utilises frequency domain parameter recovery will demonstrate greater consistency, and accurate quantitative measurements then one without this technology.

We will test this by utilising a dynamic end-tidal forcing (DEF) system to induce stepwise hypoxia in healthy volunteers, looking to see if there are any significant differences in NIRS parameters observed simultaneously by each device, and if either NIRS modality measured changes in cerebral tissue with greater consistency (with individuals undergoing identical hypoxic insults). It is important to bear in mind that this investigation was not generally comparing the fundamental FD and CW parameter recovery techniques, instead we are comparing two clinically viable ‘point of care devices’ where one has the potential advantage of FD parameter recovery. There are a variety of differences within each device that represents a confounder to addressing that specific question (e.g. source detector configuration and specific algorithmic considerations).

Each participant was exposed to isocapnic hypoxia using the technique of end-tidal forcing, which achieves accurate control of end-tidal gases by modifying the composition of inspired air on a breath-by-breath basis. This technique provides an intuitive model for studying all brain pathologies involving hypoxic changes. End-tidal carbon dioxide partial pressure (ETCO_2_) has been demonstrated to closely correlate with PaCO_2_ [12] and therefore, by fixing the end-tidal partial pressures during hypoxia we are also maintaining consistent PaO_2_ and PaCO_2_ and removing a confounding influence of ventilation on brain vascular activity. Although expensive and not widely available, this technique has proven effective in the research environment and has been used to demonstrate the significant influence of end-tidal carbon dioxide partial pressure has on brain blood flow and autoregulation [13] during functional magnetic resonance (MR) imaging. We believe that these experimental conditions will narrow the variability of cerebral physiology driven by respiratory factors.

## Methods

### Participants

Healthy Volunteers recruited from within the University of Birmingham (UK) took part in this study after providing their informed written consent. The study conformed to the Declaration of Helsinki and was approved by the University of Birmingham Research Ethics Board (Ref.: ERN_30-1031). Individuals recruited had no significant prior medical history.

### Design

A prospective healthy volunteer study, conducted under controlled laboratory conditions.

### Equipment

The NIRO 200NX (Hamamatsu Photonics, Tokyo, Japan) CW spectrometer measuring at wavelengths of 735, 810, and 850 nm was used simultaneously with an ISS OxiplexTS (ISS Inc, Champaign IL, USA) FD device measuring at wavelengths of 690 and 830 nm. Both these devices are portable, point of care devices that are commercially available to healthcare providers, and fulfil our requirements as clinically viable monitors. Their respective probes can be applied easily by individuals with minimal instruction and training. For ease of comparison and clinical translatability, a single output parameter from each device was selected. The Tissue Oxygen Index (TOI) and Tissue Hb Saturation were selected as parameters for comparison from the NIRO 200NX and ISS, respectively. It was felt that these represent popular clinically observed parameters, combining data from each Hb chromophore. Output parameters from each device were extracted at 1 Hz for the purposes of direct comparison.

To maintain continuous fixed end-tidal gaseous composition a custom made dynamic end-tidal forcing (DEF) device was employed. Gases were delivered and retrieved via a soft disposable plastic mouth piece via a filter. A nose peg was also applied to ensure no room air contamination due to inspiration through the nares.

### Procedure

Individuals underwent a step-wise DEF induced hypoxia in a supine position on an examination bed tilted upwardly approximately 25°. Simple skin cleansing and application of a device specific adhesive tape (in the case of the CW device only) was all that was undertaken in terms of surface preparation. Once the devices were in place, an elastic crepe bandage was then applied over the probes along with the addition of fabric tape to both secure the probes and minimise ambient light interference.

**Fig. 4:**
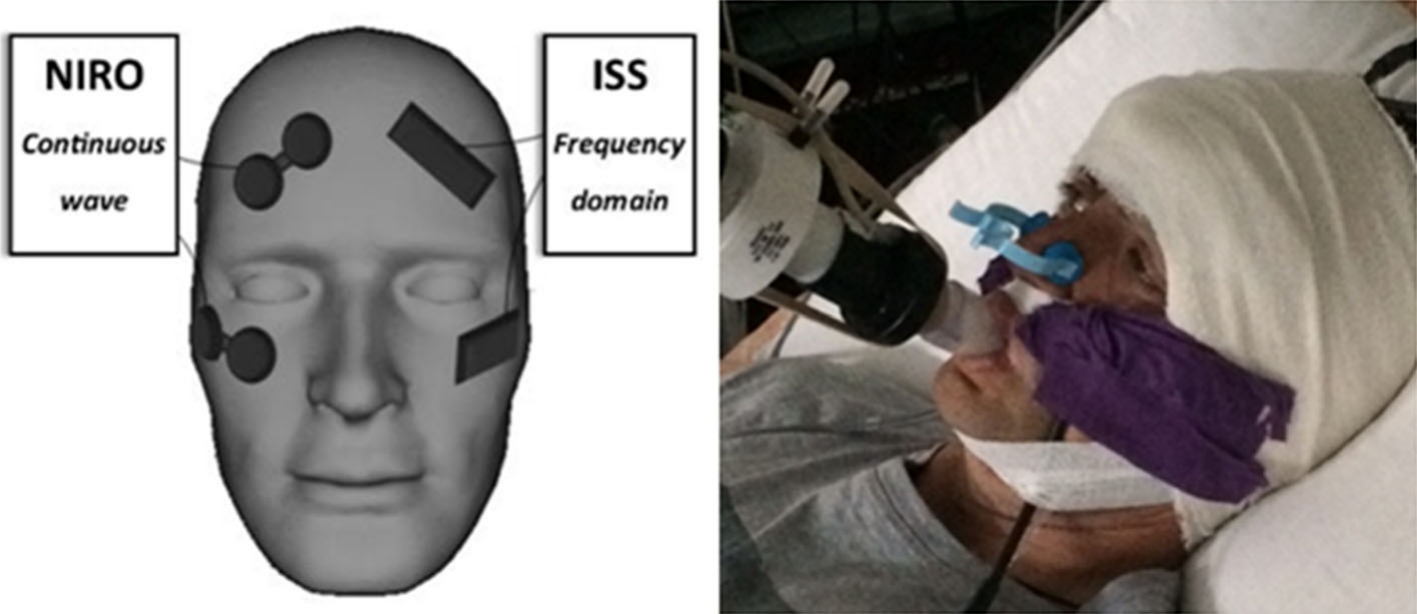
NIRS position and DEF illustrated

Each device was assigned a fixed side of observation which was maintained through the experiment (CW right, FD left) and between all participants. One probe/channel was placed on the forehead with the probe centre point located 4 cm from the midline and 3.5 cm above the superior orbital ridge (targeted at cerebral tissue) (Fig. 4). The second probe/channel of each device was placed over and in line with the zygomatic arch, targeted specifically at non-neurological (somatic tissue). The purpose of this placement was to observe any overt difference between cerebrally targeted and non-cerebrally targeted NIRS parameters during hypoxia, and if this varied between devices. This field of NIRS acquisition in health incorporates skin, bone (posterior zygoma) and muscle (pterigoid and temporalis) but no air. Anatomically this region would not normally incorporate and air cavity, and this was confirmed on a number of participant specific archive MR images.

**Fig. 5:**
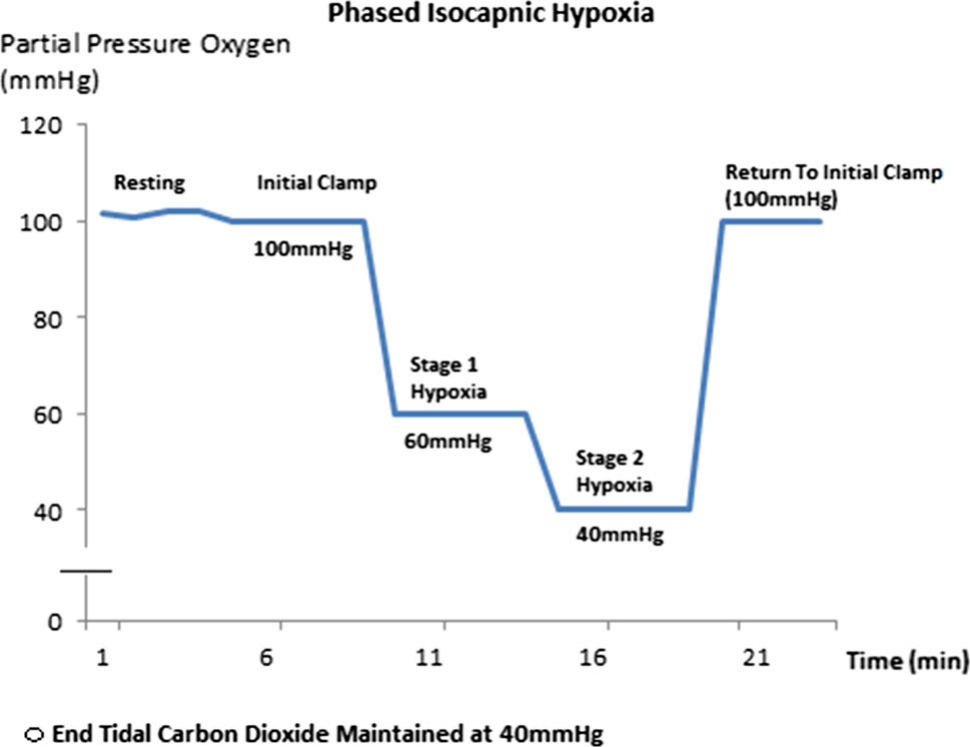
Isocapnic hypoxia protocol

An initial room air stabilisation period (ascertaining baseline expiratory gas composition) proceeded formal clamping of end-tidal gases to ensure participant comfort and consistent readings. From here, an initial end-tidal gas clamp was then applied, with end-tidal Oxygen and Carbon Dioxide clamped at 100 and 40 mmHg respectively. This was maintained for 5 min before the commencement of 2 stepwise 5-min periods of hypoxia, with isocapnia maintained. The first 5-min stage of hypoxia reduced an individuals inspired oxygen to a partial pressure of 60 mmHg, with the second reducing this further to 40 mmHg. After this the baseline clamp values were then restored and maintained for the final 5 min of the protocol (Fig. 5). Participants were asked to relax and rest during the procedure, and to breathe as naturally as possible.

Participants were continuously monitored by qualified clinical staff during the procedure, and non-verbal contact was maintained during the procedure to maintain comfort and safety.

### Data analysis

Simultaneous data obtained from both NIRS devices during the hypoxia protocol from each individual was directly compared. A variety of parameters were compared, including: baseline noise, baseline mean values, the quantity of change observed during each phase of isocapnic hypoxia and the magnitude rebound re-saturation observed when the hypoxia clamp was released. Data was compared using a variety of descriptive statistics and paired non-parametric statistical tests including Wilcoxon signed rank test. All data analysis was carried out using in house written code in a commercial software package (MATLAB (R2015a) Mathworks Inc. Natick, Ma, 2000).

## Results

### Baseline variability and noise

A total of 9 healthy individuals (6 Male) completed the isocapnic hypoxia protocol. One participant was unable to tolerate the second stage (40 mmHg) hypoxia for the full specified 5 min due to discomfort. The initial 100 s of this second stage of hypoxia was maintained and stable output parameters were observed, therefore this individual’s data was maintained for analysis.

For practical purposes during our analysis the two stages of hypoxia will be considered as a single step change in observed parameters. The main reason for hypoxia being induced in 2 stages was largely one of participant comfort.

The mean baseline parameters observed in the somatically targeted probes were consistently higher than parameters recorded by those targeted at brain tissue (77 vs 63 % CW/NIRO and 72.7 vs 61.8 % FD/ISS; *P* = 0.0078 and 0.0039, respectively). Consistently a greater level of noise was observed by the FD (ISS) NIRS device throughout the experiment (SD of baseline traces 1.37 vs 0.87 %). In terms of baseline variability, both devices demonstrated a marked variability in baseline parameters (Table 1).

**Table 1:** Table of baseline means and coefficients of variance (CV) Wilcoxon rank test significance

	Baseline TOI (%) (mean ± CV)	Hypoxia TOI (%) (mean ± CV)	Post release TOI (%) (mean ± CV)
NIRO Forehead	63.2 ± 8.4	53.4 ± 11.9	62.7 ± 11.1
NIRO Zygoma	77.0 ± 10	68.3 ± 9.54	77.9 ± 10.2
ISS Forehead	61.8 ± 9.7	54.8 ± 7.93	62.8 ± 9.9
ISS Zygoma	72.7 ± 6.8	64.1 ± 5.26	72.4 ± 6.7

### Observed changes during hypoxic clamp (Table 1)

The brain directed parameters (% Saturation/TOI) observed by both devices changed markedly on moving from the initial baseline clamp to the second stage of induced hypoxia (i.e., 9.8 and 7 % reduction from baseline for the CW/NIRO and FD/ISS devices, S.D 2.3 and 3.4 % respectively) these changes were statistically significant (Wilcoxon signed rank test *P* < 0.01 for both devices). Similar significant changes were seen in the non-neurological somatically targeted probes (*P* < 0.01 for both devices).

The changes observed during hypoxia between the neurologically targeted probes and those targeted at the zygomatic/somatic tissue were not significantly different in either probe (*P* = 0.1289 and 0.3594 NIRO and ISS respectively). This suggests a similar level of desaturation during hypoxia in both tissues, although caution must be taken in interoperating these results, this is a small sample size and a larger investigation may yield different results.

### Observed cerebral changes during release from hypoxic clamp

After release of the hypoxic clamp a substantial increase in cerebrally derived parameters was seen in both devices. This was the single largest change in parameters seen during the protocol. In both cases the changes seen by each device (from 40 to 100 mmHg) were highly significant (*P* = 0.001953 for both the NIRO and ISS), indicating once again that both devices clearly demonstrate the ability to detect the changes induced by the release of the hypoxia clamp. In all cases stable post release (100 mmHg) parameters were obtained within 40 s. A mean rise from hypoxia of 9.7 % was detected by the NIRO (Range 4.4–13.4 % median 13.2 %), with the ISS observing a slightly lower average change of 8.9 % (Range 4.6–13.7 % median 9.6 %). There was no statistically significant difference between the changes detected by either device (*P* = 0.625).

## Discussion

**Fig. 6:**
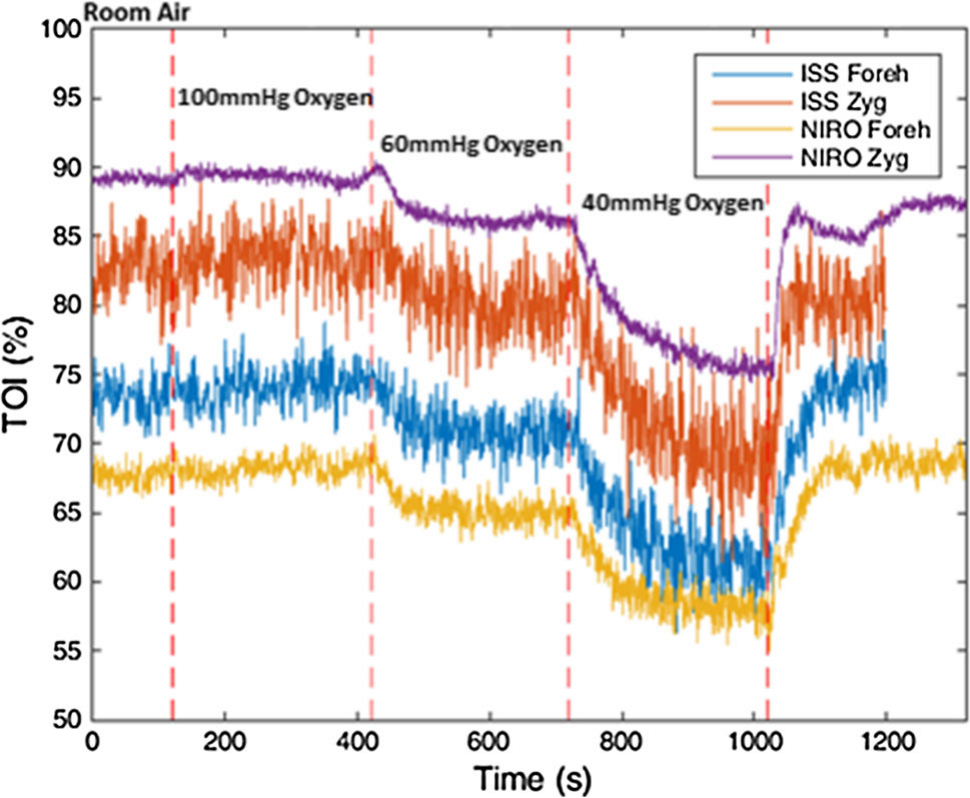
Typical participant plot, illustrating FD related noise

We have demonstrated that both of the devices tested have the ability to detect both the significant decrease in derived NIRS tissue saturation during induced hypoxia and the significant increase in these seen after release of the hypoxic clamp. Of interest was the performance of the ISS/FD device which seemed to perform in an indistinguishable way in its ‘as purchased’ form to the NIRO/CW device, despite the theoretical/technical advantage of its FD parameter recovery method. It is also noteworthy that the output parameters produced by this device were noticeably noisier (Fig. 6). This would potentially prove a disadvantage within the clinical arena and in particular acute traumatic brain injury (TBI) care, where observational conditions could be sub optimal.

The consistently lower baseline parameters observed by the probes targeted at brain tissue (compared to those targeted at somatic tissues in the facial skeleton) could be explained physiologically by the significantly lower baseline oxygen demands of the resting state bone, muscle and skin tissue compared to the brain. This is an interesting observation, highlighting the very different nature of the tissue under the respective probes and reaffirming the ability of both devices to detect cerebral activity. A limitation of this investigation is that we have not identified the specific degree by which the superficial tissues have influenced these parameters in either device, and therefore these results must be interpreted appropriately. The incorporation of different physiological conditions (hypocapnia), or other respiratory manoeuvres may potentially help quantify this in future studies.

The level of noise observed in the output parameters of the ISS/FD device was noticeably (and consistently) greater than that seen from the NIRO/CW. This may be explained by the highly sensitive phase measurements made by this frequency-domain model, detecting moment- to- moment changes in physiology and as output data are at a modest 1 Hz this would intuitively lead to a more noisy trace (Fig. 6). In addition the FD/ISS device utilised fibre optic light transfer which is then amplified within the device by a photo-multiplication device, as opposed to the NIRO/CW device which detects light from the tissues directly via surface photodiodes. These differences in light detection methods may also contribute to the levels of noise observed in output parameters.

For the purposes of clinical application, the coefficient of variance of baseline cerebral parameters seen (8.4 and 9.7 % for the NIRO and ISS respectively) is greater than the reduction seen in the case of 3 subjects in the case of the NIRO, and 4 subjects in the case of the ISS. This indicates that the baseline parameters obtained from either device is not sufficiently consistent to be utilised for clinical purposes.

The outcomes of this investigation cannot be extrapolated to FD-NIRS technology in its entirety, but in this tested ‘point of care’ configuration. Franceschini et al. [14] reported accurate quantitative measurements in a cohort of healthy neonates. This utilised a modified ISS/FD device emitting an additional 4 wavelengths of light (6 vs 2) from each source diode, increasing system redundancy and providing clinically accurate output parameters. Due to these examples in the established literature we are cautious to apply our findings to FD-NIRS generally. Therefore, FD certainly has the potential to enhance the clinical application of NIRS, despite no clear indication of an advantage in our findings.

The focus of this investigation was on the ‘as supplied’ unmodified form of the devices, as modification of these devices requires specialist knowledge and is potentially not immediately translatable to clinical care. With this in mind, we found that in tested form our FD device did not demonstrate distinguishably different abilities from the CW NIRO 200NX.

In terms of clinical application, both devices could potentially be utilised to observe changes in cerebral physiology, however baseline parameters (quantitative measurements at the commencement of observation) are highly variable and not useful for clinical decision making. These limitations are widely acknowledged [15–17] and the significant variability observed in studies contemporaneously comparing NIRS to functional MR imaging [18]. The obvious potential benefits of FD-NIRS were recognised by Calderon-Arnulphiet et al. [19], when they undertook an investigation into to abilities of a device (similar to device employed by our investigation) to detect ischemic changes during vascular neurosurgical procedures. They reported satisfactory detection rates of ischemia. However, their methodology focused primarily on the relative changes to initial baseline (although making reference to absolute chromophore quantity).

Both probes detected significant changes in parameters during the induction of hypoxia. This combined with the significantly higher baseline saturation seen in the somatic facial tissue indicates that both devices are capable of reliably detecting the change in specific cerebral oxygenation induced, and either one could be used as an effective monitoring tool (within the tested context). The changes we observed were not unexpected as we anticipated a marked change in cerebral saturation after inducing such a profound hypoxia equivalent to what would be experienced at the summit of the world’s tallest mountains (5000 meters).

Within the specific context of acute TBI care (particularly within a pre-hospital context), decisions regarding brain health and the need for intervention are often required soon after patient contact. Should NIRS be utilised in the clinical decision making process in this tested form little could be derived from the initially extracted parameters. Certainly a trend could be established as to the direction of change in parameters after a period of observation; however without initial validation/calibration (with invasive monitoring or axial imaging) this would not be useful in immediate management. When a baseline level of function/brain health is known (as in cardiac bypass surgery/cardiopulmonary resuscitation) the uncertainty regarding baseline parameters becomes less important and the change in trends observed becomes clinically useful [20, 21]. For any NIRS device to be employed suitably within a TBI context it must be able to clearly distinguish grossly abnormal parameters (such as those related to significant hypoxia) from the expected normal observations of the majority of individuals.

Currently, true quantitatively accurate NIRS parameters have only been obtained within breast tissue [22], utilising a diffuse optical tomographic (DOT) array. These techniques involve large (clinically impractical) arrays of sources and detectors and parameters are not able to be effectively reconstructed in real time, and still fail to provide absolute quantification in the context of cerebral tissue monitoring [23]. A significant limiting factor in all currently available (clinically viable) NIRS devices is knowledge of spatial priors [24] (specific dimensions of tissue layers), and incorporation of patient specific atlas (MRI) based data may aid in the development of future clinically viable devices that can provide a better quantitative measurement of chromophore concentration within cerebral tissue [25].

## Conclusion

Although FD-NIRS clearly has many advantages over continuous-wave devices, our investigation demonstrated that the device tested performed indistinguishably from a similar clinically available ‘as supplied to clinicians’ CW device within the context of detecting induced hypoxia. The variability seen in baseline parameters between individuals (in this limited investigation) by FD-NIRS is similar to that of CW devices, and therefore does not offer any direct advantage of CW devices in the acute assessment setting.
